# Ecological risk and spatial distribution, sources of heavy metals in typical purple soils, southwest China

**DOI:** 10.1038/s41598-024-59718-9

**Published:** 2024-05-18

**Authors:** Cang Gong, Licheng Quan, Wenbin Chen, Guanglong Tian, Wei Zhang, Fei Xiao, Zhixiang Zhang

**Affiliations:** 1 Research Center of Applied Geology of China Geological Survey, Chengdu, 610039 China; 2 Key Laboratory of Natural Resource Coupling Process and Effects, Beijing, 100055 China

**Keywords:** Purple soils, Ecological risk, Spatial distribution, Heavy metals, Geographical detector, Ecology, Environmental sciences

## Abstract

The identification and quantification of the ecological risks, sources and distribution of heavy metals in purple soils are essential for regional pollution control and management. In this study, geo-accumulation index (I_geo_), enrichment factor (EF), pollution index (PI), potential ecological risk index (RI), principal component analysis (PCA) model and geographical detector (GD) were combined to evaluate the status, ecological risk, and sources of heavy metals (HMs) in soils from a typical purple soil areas of Sichuan province. The results showed that the average contents of As, Cd, Cr, Cu, Hg, Ni, Pb and Zn in purple soil were 7.77, 0.19, 69.5, 27.9, 0.077, 30.9, 26.5 mg/kg and 76.8 mg/kg, and the I_geo_, EF and RI of topsoil Hg and Cd in designated area was the highest, and the average contents of Hg and Cd in topsoil were obviously greater than respective soil background value in Sichuan province and purple soil. The hot spots for the spatial distribution of 8 HMs were mainly focused in the southwest and northeast of the designated area, and there were also significant differences for 8 HMs distribution characteristics in the profile soil. Cu comes from both anthropogenic and natural sources, Zn, Ni and Cr mainly come from natural sources, but As, Pb, Hg and Cd mainly derived from human activities. GD results showed that soil texture (X_18_), altitude (X_4_), total nitrogen (TN), clay content (X_3_), sand content (X_2_) and silt content (X_1_) had the greatest explanatory power to 8 HMs spatial differentiation.This study provides a reference for understanding the status and influencing factors of HM pollution in typical purple soil, and lays a theoretical foundation for the environmental treatment of purple soil in China.

## Introduction

Soil is a precious natural resources, human survival and agricultural production are inseparable from clean soil. Accompanied by rapid development of agriculture, industrialization and urbanization, the problem of soil pollution has become increasingly prominent^1,2^. Global soil heavy metal (HM) pollution accidents occur frequently and the form is becoming more and more serious. Soil HM contamination has become one of the serious eco-environmental issues faced by the development of various countries^3,4^. Soil heavy metals (HMs) accumulated will have a negative impact on soil nutrient cycling^5^ and crop yield and quality^6^. Soil HMs can enter body through skin contact and inhalation^7^or by the food chain^8^. Generally speaking, in nature, soil HMs mainly come from parent materials of soil^9^, and natural HMs mainly exist in the form that plants are difficult to use, and there is a low ecological risk^10^. Human activities, such as agricultural fertilization, industrial activities and vehicle emissions can increase the accumulation of HMs in soil^11^. Anthropogenic HMs often take high biological activity, are easy to be absorbed and utilized by plants, and have high ecological risks, and aggravate HMs spatial variability in soil. It is the key to effective deal with soil HM pollution, trace its source and explore the factors that affect the spatial differentiation characteristics of soil HMs. Last 10 years, many researches and analysis have been conducted both domestically and internationally on the driving factors of soil HM pollution^12–17^.

In recent years, the evaluation indexes of soil HM pollution, including factor of enrichment (EF), index of potential ecological risk (RI) and index of geological accumulation (I_geo_) has been widely applied in practice^18^. The method of contaminant source division can be divided into identification of pollution source and quantification of pollution source. Pollution source identification is usually calculated by the cluster analysis, principal component analysis^19^ and geographic information system^20^, while the quantification of pollution sources is realized by receptor models, such as positive matrix factorization, balance of chemical mass, and geographic detector, which is extensively used because they can quantitatively display the contribution of different pollution sources in many researches^4,21–23^. Multivariate statistical analysis, geostatistical models^24^ and some comprehensive methods have gradually improved the study of the sources and spatial differentiation of HMs in soils. Accordingly, various evaluation indexes were applied to assess soil HM contaminant, such as I_geo_, EF, RI and pollution index (PI)^25^.

Purple soil is usually classified as regosol or entisol^26^. A series of red or purple rocks from the Triassic to Cretaceous period formed the purple soil^26^. A large amount of this type of soil is distributed in the Sichuan Basin, which is one of the greatest agricultural areas in southwest China. With the serious trouble between the land resources limitation and the population surge, more and more purple soil was used into agricultural soil, and part purple soil was developed for production of intensive agricultural because its rich mineral nutrition. Most of the existing studies focus on the background value, baseline, content^27^, distribution^28^ and migration characteristics^29^ of HMs in purple soil, and there are few studies on the sources, ecological risk and spatial differentiation of HMs in purple soil. In particular, there are few reports on the potential ecological risks and driving factors of HMs in purple soil. This study aims to (1) assess the concentration and pollution levels of HMs in typical purple soils; (2) identify the main sources of HMs in typical purple soils; (3) assess the main drivers of HM pollution in typical purple soils based on the GeoDetector model; and (4) assess the potential ecological risks of HMs in typical purple soils. The research results can provide important theoretical and practical reference for the source identification, risk assessment and comprehensive treatment of HM pollution in purple soil, and will lay a theoretical foundation for environmental management and regional sustainable development of purple soil in China.

## Materials and methods

### Research area

The research area was situated in the east of Sichuan Province, longitude 105°56′-107°19′ E, latitude 30°01′-30°52′ N, with an area of 6339 km^2^ and a total population of 4.7 million. The topography of the research area is east high and west low, and the zoning characteristics are obvious. The research area was situated in the humid monsoon climate area of the middle subtropics, with a warm climate, abundant heat and abundant rainfall, an annual average precipitation is 1054.46 mm-1512.45 mm, an annual mean temperature is 16 ℃. The territory of 210, 212, 305, 318 national highways and 203, 304, 18 provincial highways and county, township, village roads crisscross, the highway network extends in all directions. The Jialing River and the Qujiang River cross the border.

### Sampling and analysis

**Figure 1:**
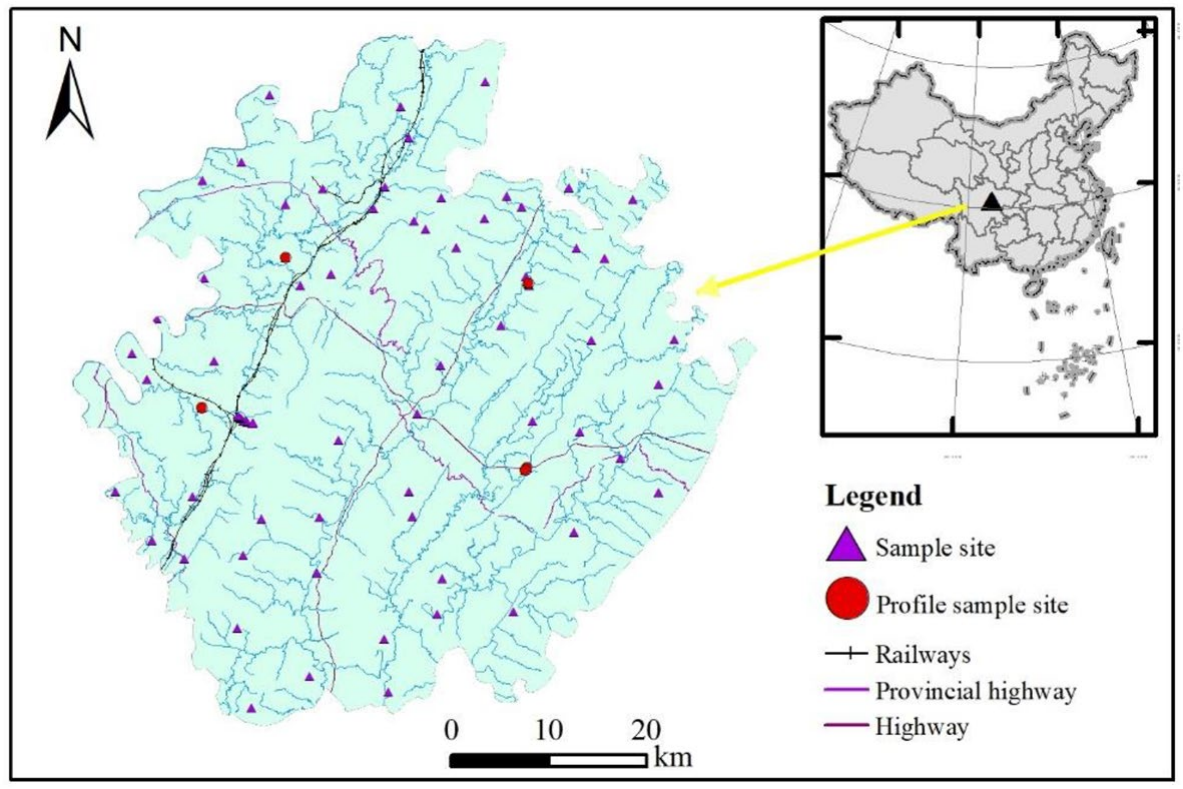
The location of research area and sampling sites.

Field sampling to be completed in 2022. A total of 73 topsoil samples (0–20 cm) were collected according to the specification of land quality geochemical assessment (DZ/T 0295–2016)^30^. Sampling sites were displayed in Fig. 1. The sampling points were uniformly arranged in a 4 km^2^ sampling grid, and the distance between each sampling point was required to be greater than 2 km. 3–5 multi-point collections within 100 m around the sampling point are combined into one sample, and the original weight of the combined sample is greater than 1 kg. Locate sampling points with portable GPS. 5 profile sampling points PMA, PMB, PMC, PMD and PME in the study area, with a profile depth of 1 m, and one sample is collected for each 20 cm.

C_org_ was measured by volumetric method, N was measured by combustion infrared method, atomic fluorescence method was used measure the content of Hg and As, Cu, Pb, Ni, Zn, Sc, Cr, P, Cd and K were measured by x-ray fluorescence spectroscopy (XRF), inductively coupled plasma mass spectrometry (ICP-MS) and inductively Coupled Plasma Optical Emission Spectrometer (ICP-OES). The quality of analysis and testing was controlled by means of inserting national first-level soil standard materials, repeatability inspection, abnormal point inspection, and blank test.

### Geo-accumulation Index (I_geo_)

The I_geo_ method can be used to compare the concentration of different soil HMs and their pollution degree^31^.

1 $${\text{I}}_{{{\text{geo}}}} = {\text{ log}}_{{2}} \left( {\frac{{C_{i} }}{{K \times B_{i} }}} \right)$$

where I_geo_ was soil accumulation index of HM *i*; C*i* was measured value of soil HM *i*; B*i* was guideline value, and background value of profile soil was selected (Table 1); k was the correction coefficient, generally 1.5. The pollution degree of I_geo_ can be divided into seven grades: < 0 (unpolluted), 0–1 (mild contaminated), 1–2 (moderate contaminated), 2–3 (moderate-heavy contaminated), 3–4 (heavy contaminated), 4–5 (heavy-extreme contaminated) and > 5 (extremely heavy contaminated).

**Table 1:** Descriptive statistical results of topsoil composition.

Composition	Unit	Min	Max	Mean	S.D	Median	CV (%)	Background values of Sichuan Province^37^	Background values of purple soils^37^	Purple soil in other area			Threshoid Values^a^ (5.5 < pH ≤ 6.5)
										Sichuan Nanchong^38^	Sichaun Xichang^39^	Chongqing^40^	
As	mg/kg	1.61	42.7	7.77	7.32	5.08	94.3	10.4	9.4	11.09	6.29	7.26	40
Cd	mg/kg	0.038	0.51	0.19	0.11	0.16	56.3	0.079	0.094	0.23	0.08		0.3
Cr	mg/kg	34.8	151	69.5	17.4	66.1	25.1	79	64.8	64.99	92.74	52.8	150
Cu	mg/kg	4.31	108	27.9	16.85	25.4	60.3	31.1	26.3	35.26	20.5	28.8	50
Hg	mg/kg	0.010	0.73	0.077	0.089	0.057	116.5	0.061	0.047	/	0.029	0.036	1.8
Ni	mg/kg	9.60	73.3	30.9	11.0	30.6	35.5	32.6	30.7	38.77	39.48	37.4	70
Pb	mg/kg	15.0	76.0	26.5	7.66	25.9	28.9	30.9	27.7	28.98	25.78	23.5	90
Zn	mg/kg	25.6	121	76.8	19.9	82.1	25.9	86.5	82.8	100.65	89.82	82	200
TC	%	0.12	26.2	1.18	3.04	0.68	258	/	/	/	/	/	/
Corg	%	0.11	13.0	0.90	1.53	0.58	170	1.91	0.75	/	/	/	/
TN	mg/kg	293	2031	830	407	738	49.1	/	/	/	/	/	/
TP	mg/kg	152	922	442	177	405	40.1	/	/	/	/	/	/
TS	mg/kg	57.5	1314	189	174	140	92.0	/	/	/	/	/	/
TK	%	0.98	3.24	1.94	0.50	1.98	25.8	2.02	2.00	/	/	/	/
Sc	mg/kg	5.72	28.1	13.0	3.65	12.9	28.0	12.01	11.65	/	/	/	/
Silt grain	%	9.3	49.7	30.8	9.96	31.2	32.4	/	/	/	/	/	/
Sand grain	%	4.0	83.6	42.5	18.15	43.5	42.7	/	/	/	/	/	/
Clay particle	%	3.3	60.8	26.8	11.60	24.1	43.3	/	/	/	/	/	/

^a^Soil contamination risk screening values (GB 15,618–2018).

### Enrichment Factor (EF)

EF was a useful index to distinguish between human activities or natural sources of HMs. EF was calculated^32^:

2 $${\text{EF}} = \left[ {{\text{M}}_{i} /{\text{M}}_{Sc} } \right]_{{\text{S}}} /\left[ {{\text{M}}_{i} /{\text{M}}_{Sc} } \right]_{{\text{B}}}$$

where [M_*i*_/M_*Sc*_]_S_ was the content ratio of HM *i* to Sc in samples, while [M_*i*_/ M_*Sc*_]_B_ was the ratio of purple soil background values. Sc was a trace element, and has no significant anthropogenic sources, so Sc was selected as guideline element^32^. Generally, according EF value the soils can be classified as six enrichment grades: < 1 (minimal), 1–2 (mild), 2–5 (moderate), 5–20 (significant), 20–40 (very high), and ≥ 40 (extremely high).

### Pollution index (PI) and synthetic pollution index (SPI)

For evaluate soil HMs contamination level, PI and SPI were calculated:

3 $${\text{PI }} = \frac{{C_{i} }}{{S_{i} }}$$

4 $${\text{SPI }} = \sqrt {\frac{{\left( {\frac{{C_{i} }}{{S_{i} }}} \right)_{max} + \left( {\frac{{C_{i} }}{{S_{i} }}} \right)_{ave} }}{2}}$$

where PI was element *i* contamination index, SPI was overall score of each HM to the composite contamination. S_*i*_ was the valuation criterion of element *i*, and the national control thresholds were selected as criterion (Table 1). According PI and SPI values the soils can be classified as 5 contamination categories: < 0.7 (safety), 0.7–1 (alert), 1–2 (low), 2–3 (moderate) and ≥ 3 (severe)^4^.

### Potential ecological risk factor (ER) and Potential ecological risk index (RI)

ER was applied to assess potential ecological risk of individual soil HM^33^, ER was calculated:

5 $${\text{ER}} = {\text{TR}}_{i} \times {\text{PI}}_{i}$$

where TR_*i*_ was HM (*i*) toxic-response factor of Hg (40), Cd (30), As (10), Pb (5), Cu (5), Cr (2) and Zn (1)^33^, TR of Ni was 5^34^. PI_*i*_ was HM (*i*)contamination index of. The ER classes can be divided into five potential ecological risk grades: < 40 (low), 40–80 (moderate), 80–160 (considerable), 160–320 (high) and ≥ 320 (very high)^35^.

RI was a method to evaluate soil multi-element ecological risk. RI was calculated^33,35^:

6 $${\text{RI}} = \mathop \sum \limits_{i = 1}^{n} ER = \mathop \sum \limits_{i = 1}^{n} \left( {{\text{TR}}_{i} \times {\text{PI}}_{i} { }} \right)$$

where $\mathop \sum \limits_{i = 1}^{n} ER$ was HM (*i*) potential ecological risk factor, *n i* the number of HMs. RI classes can be divided into four potential ecological risk grades: < 150 (low), 150–300 (moderate), 300–600 (considerable) and ≥ 600 (very high)^35^.

### Geographical detector (GD)

Factor detector was one of the four methods for GD^36^, applied to determine spatial differentiation of dependent variables and the ability of their corresponding variables to illustrate the effect of dependent variables, evaluated by the value of *q*:

7 $$q = 1 - \frac{{\mathop \sum \nolimits_{h = 1}^{L} N_{h} \sigma_{h}^{2} }}{{N\sigma^{2} }} = 1 - \frac{SSW}{{SST}}$$

where *h* = 1, …, L was the classification number of the independent variable X, N_h_ and N were the classification h and the number of units in the whole region, $\sigma_{h}^{2}$ and $\sigma^{2}$ were variance of the dependent variable Y in the classification *h* and the region. SST and SSW represent the total variances of all categories of the independent variable X and the sum variance in the region. The range of *q* was [0,1]. The larger *q* value, the greater impact of the independent variable X on the dependent variable Y.

### Factor selection and data processing

Select soil properties (organic carbon (C_org_), silt content (X_1_), total carbon (TC), total potassium (TK), total nitrogen (TN), clay content (X_3_), total phosphorus (TP), sand content (X_2_) and total sulfur (TS)), topographic factors (altitude (X_4_), slope (X_5_) and aspect (X_6_)), distance factor (distance from railways (X_7_), distance from highway (X_8_), distance from provincial highway (X_9_), distance from county road (X_10_), distance from rural road (X_11_), distance from village road (X_12_), distance from the river (X_13_), distance from lakes and reservoirs (X_14_), distance from urban area (X_15_), distance from the town (X_16_) and distance from the village (X_17_)), soil texture (X_18_) and land use type (X_19_) 25 factors. When using GD to analyze the impacting factors, all independent variables must be converted to type variables and the dependent variable must be a numerical variable. In this research, natural breakpoint method was applied to classify the impacting factors. Analysis of correlation and descriptive statistical analysis of the data was carried out by SPSS26.0, sampling map and spatial distribution map were drawn by ArcGIS10.8, mapping was completed by Origin2019, and GD was completed by GeoDetector software (http://www.geodetector.org/).

## Results and discussion

### Basic properties of topsoil in study area

HMs contents and physicochemical properties in topsoil of study area were displayed in Table 1. Mean concentrations of TC, Corg, TN, TS, TP and TK were 1.18%, 0.90%, 830 mg/kg, 189 mg/kg, 442 mg/kg and 1.94%, and the mean contents of silt grain, sand grain, clay particle were 30.8%, and 42.5%, and 26.8%.

Average concentrations of Cd, As, Cr, Hg, Cu, Ni, Zn and Pb were 0.19, 7.77, 69.5, 0.077, 27.9, 30.9, 76.8 mg/kg and 26.5 mg/kg. Average contents of Cd and Hg larger than the Sichuan province soil background values^37^, compared with purple soil background values^37^, average contents of Hg, Cd, Cr, Cu and Ni were greater than the values of background. Compared with the risk screening standard value for soil (GB15618-2018), average contents of all HMs were less the contamination risk screening value, but the maximum As, Cu, Cr, Cd and Ni contents were 1.07, 2.16, 1.01, 1.69 and1.05 times higher than their corresponding pollution risk screening value. The coefficient variation was proportional to the degree of interference from external factors such as human activities^22^, the coefficient variation of Hg and As were 116.5% and 94.3% in study area indicates that they may be affected by some external interference factors. In comparison with other purple soil areas except that the content of Zn was higher than that of purple soil in Sichuan Nanchong, the contents of other HMs were lower than those in purple soil in Nanchong, Sichuan^38^. Contamination of Hg, As, Pb, Cd and Cr were greater than their corresponding purple soil values in Sichaun Xichang^39^, and the contents of As, Cd, Hg, Cu and Pb were larger than respective purple soil values in Chongqing^40^.

### Soil HMs spatial distribution features

**Figure 2:**
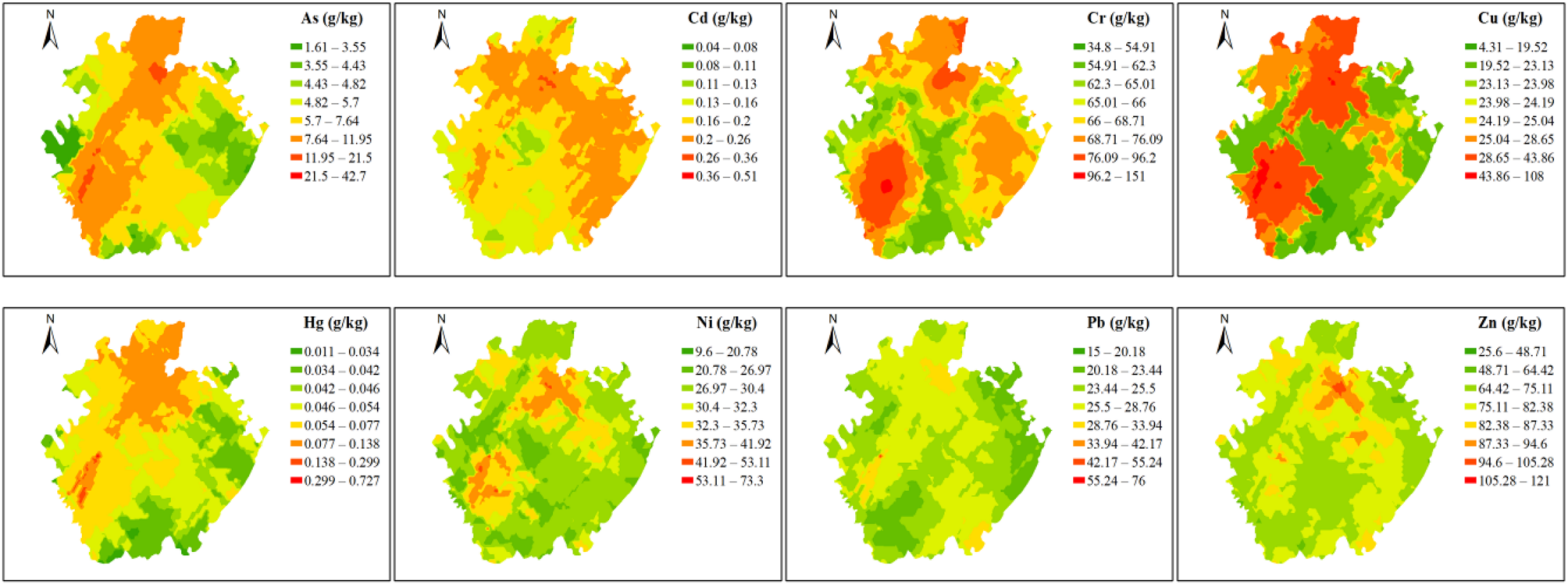
HMs spatial distribution in the topsoil.

Figure 2 shows HMs spatial distribution in purple soil of research area. As a whole, soil HMs spatial distribution was significant various from the south to north of this research area. It can be seen that the large value regions of Cu, As, Zn, Hg, Pb, Ni and Cr were mainly distributed in northeast and southwest regions. The hotspots of large Cd distributed in north and northeast areas.

### Profile distribution characteristics of soil HMs

**Figure 3:**
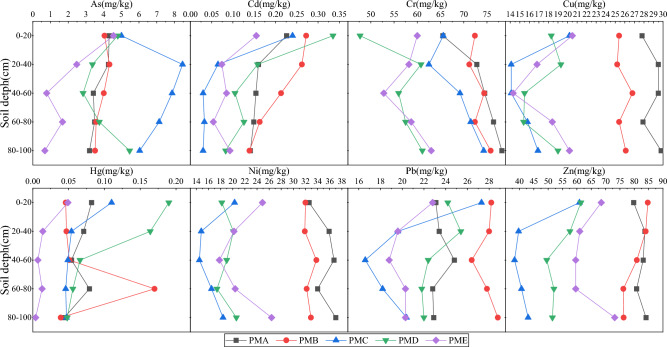
HMs profile distribution in the research region.

The average HMs contents in the purple soil profiles were showed in Fig. 3. The content of Cr was then increased greatly versus depth and minimum value in the surface soil in all profile sampling points. The distribution patterns of Cd and Hg in soil were opposite. For As and Pb, fluctuations changed with increasing soil depths. For Cu, Ni and Zn, at sampling sites of PMC, PMD and PME decreased at first and then increased with the increase of depth, while at sampling sites of PMA and PMB did not change significantly with depth.

### Evaluation of environmental risk

**Figure 4:**
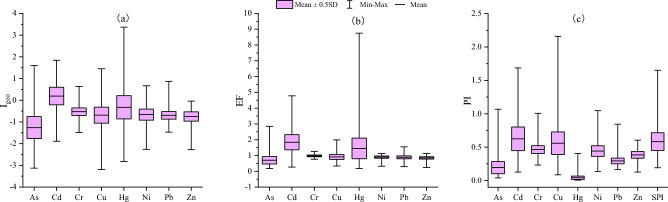
The I_geo_ (**a**), EF (**b**), PI (**c**) and SPI (**c**) for HMs in purple soils of study area.

The results of I_geo_, EF, PI and SPI were presented in Fig. 4. The average I_geo_ (Fig. 4a) values followed the descending sequence: Cd > Hg > Cr > Ni > Cu > Pb > Zn > As, mean I_geo_ values of other 7 HMs except Cd were < 0, indicating that purple soil in research area was unpolluted by other HMs, but soils were unpolluted to moderately polluted by Cd. Among all HMs, Hg and Cd had the greatest mean values of EF (1 < EF < 2) (Fig. 4b), meaning that Hg and Cd were mild enrichment in purple soil, the highest EF of Hg and Cd were 8.75 and 4.78. Mean values of EF for other HMs were below 1, showed the minimal enrichment. Although the largest PI (Fig. 4c) values of Cu, Cd, As, Ni, Cr, Pb, Zn and Hg were 2.16, 1.69, 1.07, 1.05, 1.01, 0.84, 0.61 and 0.40, the PI means of all HMs were below than 0.7. It means that research area soil generally has no significant pollution, but there is a phenomenon of point source pollution. The mean SPI values (0.58 ± 0.26) for all HMs, meaning that the soil has no significant pollution with all HMs.

### Evaluation of ecological risk

**Figure 5:**
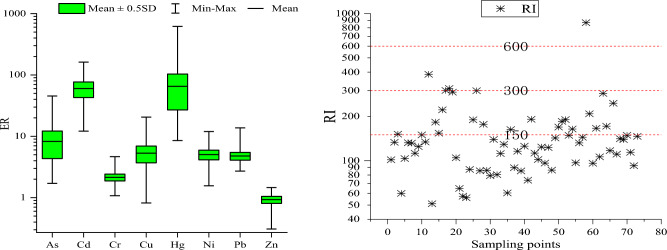
The ER and RI for HMs in soils study area.

The values of ER and RI were given in Fig. 5. Average ER values followed the descending order: Hg (65.2) > Cd (59.9) > As (8.26) > Cu (5.31) > Ni (5.04) > Pb (4.78) > Cr (2.15) > Zn (0.93), Hg and Cd highest mean ER values (40 < ER < 80), meaning that soil Hg and Cd potential ecological risk were moderate, other HMs potential ecological risk were low with mean ER values < 40. The samples with ER values higher than 40 of Cd, Hg and As respective accounted for 65.8%, 58.9% and 2.74%, indicating that Cd and Hg in research area has significant ecological risks, As has certain ecological risks, the ecological risk of other HMs was low. Mean value of RI was 152 and the range of RI values were 51.0 to 868, the samples with RI values higher than 150 accounted for 31.5%, indicating that demonstrating moderate ecological risk in this research. This was similar to the ecological risk estimation results of HMs in Xichang purple soils by Li^39^.

### Multivariate statistical methods

#### Correlation analysis

**Figure 6:**
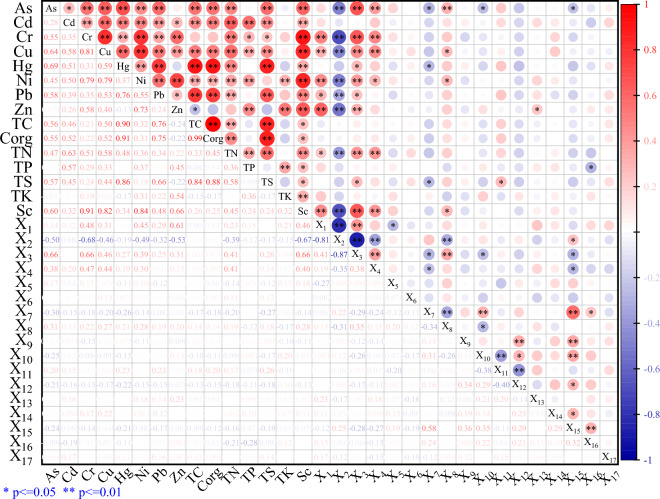
Pearson correlation coefficient of purple soil HMs and impact factors.

Correlation analysis results were displayed in Fig. 6. The results displayed that there was obviously correlation among most HMs, but interestingly, there was no significant correlation among As-Zn and Hg-Zn. Among the influencing factors of soil properties (TC, Corg, TN, TP, TK, TS, Sc, X_1_, X_2_ and X_3_), 8 HMs were significantly correlated with 6–9 of them, indicating that soil properties had significant effects on all HMs except Ni. Among the topographic factors (X_4_, X_5_ and X_6_), only As, Cr, Cu, and Ni showed significant positive correlation with X_4_. Among the distance factors (X_7_, X_8_, X_9_, X_10_, X_11_, X_12_, X_13_ X_14_, X_15_, X_16_ and X_17_), only As showed a significant positive correlation with X_8_, but an obviously negative correlation with X_7_, X_10_ and X_15_, a obviously negative correlation between Ni and Cu with X_8_, and a obviously negative correlation between Hg with X_7_. It means that the distance factor has a inevitable effect on HMs in purple soil. Generally, 8 kinds of HMs are greatly affected by natural factors, but they are affected by some external sources, which was consistent with the previous research^41,42^.

#### Principal component analysis (PCA)

The PCA results of PTEs in soils were showed in Table 2. The test value p less than 0.0001 of Bartlett's sphericity and KMO score of 0.753 meaning that the dataset was suitable for PCA. The eigenvalues of two factors (3.19 and 2.89), the cumulative contribution rate was 76.1%, which could basically explain the information contained in the purple soil HMs.

**Table 2:** PCA results of HMs in purple soil.

Variable	Hg	As	Pb	Cd	Zn	Ni	Cr	Cu	Eigenvalues	% of variance	Cumulative %
PC1	**0.966**	**0.803**	**0.790**	**0.521**	− 0.136	0.392	0.361	**0.647**	3.19	39.9	39.9
PC2	− 0.030	0.198	0.229	0.376	**0.912**	**0.870**	**0.808**	**0.647**	2.89	36.2	76.1

Significant values are in [bold].

The first principal component (PC1) had strong positive loadings with Hg (0.966), As (0.803), Pb (0.790) and Cd (0.521), and explained 39.9% of the sum variance. According to the results of correlation analysis, there was a very obviously correlation (p < 0.01) between As-Hg, As-Pb, Hg-Cd, Hg-Pb and Pb–Cd, and a significant correlation (p < 0.05) between As with Cd, indicating that the four HMs probably came from the same source, which was consistent with the results of PCA. The mean values of Pb, Hg and Cd were greater than their respective Sichuan Province soil background values and purple soil background values. Although the content of As was less than the value of background, its variation coefficient was as high as 94.3%, indicating that As was disturbed by pollution from external sources that cannot be ignored. Combined with the spatial distribution map (Fig. 2), the great content areas of As, Hg, Pb and Cd are mainly concentrated in southwest and northeast of research region. The central part of study area is a densely populated and traffic-intensive area, which is affected by huge domestic sewage discharge, industrial wastes, motor vehicle exhaust emissions, pesticides and chemical fertilizers and other external pollution sources. We determine PC1 as the source of human activity pollution.

The second principal component (PC2) explained 36.2% variance contribution rate, the strong loadings on Zn (0.912), Ni (0.870), Cr (0.808) and Cu (0.647). Although the contents of Cr, Ni and Cu except Zn were slightly higher than corresponding purple soil background values in China, the four HMs were all less than the corresponding Sichuan Province soil background values (Table 1). These four HMs showed a very important positive correlations with each other (r > 0.58, p < 0.01) except Cu–Zn (r = 0.40, p < 0.01). Also, these four HMs exhibited lower EF and I_geo_ values. Related studies show that Cr, Zn, Ni and Cu are mainly affected by soil parent materials^43^. They were mainly controlled by natural sources. PC1 can be judged to be a natural source. It should be noted that Cu also has equal loading on PC1 (0.647), indicating that Cu has the possibility of multiple sources. Previous research on this area pointed out that soil parent material plays an serious role in soil Cu enrichment, and the use of chemical fertilizers affects Cu enrichment to a certain extent^44^.

### Influencing factors affecting the spatial heterogeneity of HMs according to GD

**Figure 7:**
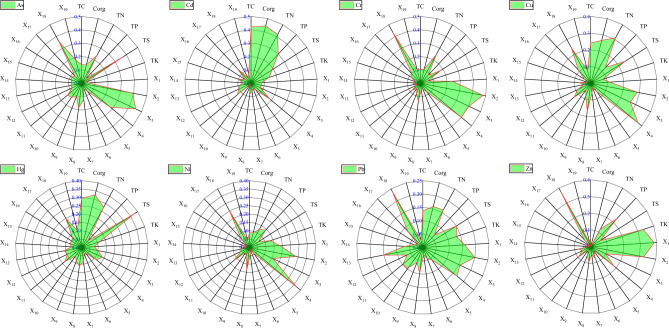
The *q* value of influencing factors affecting spatial heterogeneity of soil HMs in study area.

The geographical detection results of 25 factors for 8 kinds of HMs were displayed in Fig. 7. There was certain discrepancy in the explanation power of various factors for the 8 HMs, but overall, X_18_, X_4_, TN, X_3_, X_2_ and X_1_ have great explanatory power for each HM content spatial distribution. The first impacting factor of As was X_3_ (0.450), followed by X_2_ (0.240), TS (0.372) and X_18_ (0.360), followed by X_14_ (0.280) and TN (0.236). The first influencing factor of Cd was Corg (0.443), followed by TC (0.422) and TN (0.417), TP (0.299), X_4_ (0.189) and TS (0.188) also had significant influence. The first, second and third influencing factors of Cr were X_2_ (0.433), X_18_ (0.433) and X_3_ (0.403). X_4_ (0.399), TN (0.246) and X_1_ (0.242) also has great influence. The main influencing factors of Cu were X_4_ (0.37) and TN (0.311), and X_2_ (0.286), Corg (0.265), X_3_ (0.264), TS (0.247), X_18_ (0.243) and TC (0.242) were also important factors impacting the spatial differentiation of Cu content. The first impacting factor of Hg was TS (0.376), followed by Corg (0.326), and TC (0.293), TN (0.274), and X18 (0.201) also has significant effects. The primary influencing factor of Ni is X_4_ (0.368), followed by X_2_ (0.287) and X_18_ (0.253), followed by X_1_ (0.184) and X_3_ (0.156). The first, second, and third influencing factors of Pb are X_18_ (0.238), X_2_ (0.200), and X_4_ (0.168). TN (0.16), X_1_ (0.158), and Corg (0.158) also has significant effects. The five factors that have the greatest impact on the spatial differentiation of Zn content were X_1_ (0.383), X_18_ (0.374), TK (0.334), X_2_ (0.326), and TP (0.229).

The ranking of the impact factors on different HMs varies, revealing the heterogeneity of the mechanisms of HM changes. A large area of crops was planted in research area, and feeding, irrigation, and spraying pesticide directly impact soil physical and chemical properties, accompanied by the introduction of HMs^45^. Changes in soil physical and chemical properties directly affect soil HMs activity^46^, migration and transformation^47^. Correlation analysis also showed that TC, Corg, TN, TP, TK, TS, X_1_, X_2_, X_3_ and X_18_ were significantly correlated with one or more HMs, so soil physical and chemical properties were important indicators affecting the spatial distribution of HMs. Topographic factors reflect that the soil HMs spatial distribution was affected by natural factors, and the influence process is slow^48^. The altitude (X_4_) of research area has a significant impact on 8 HMs spatial distribution, especially on Cu and Ni. Many previous studies have also shown similar conclusions^49,50^. Interestingly, comparing the Pearson correlation analysis, it was found that there was consistency and difference in the results between Pearson correlation analysis with geographic detection analysis. Consistency, such as the significant relevance between altitude and As, Cr, Cu, and Ni, also has important impact on their spatial distribution; Differences, such as correlation analysis, showed no significant correlation between altitude and Cd, Hg, Pb, and Zn. Geographic detection analysis showed that altitude had a significant impact on the Cd, Hg, Pb, and Zn spatial distribution. It was because GD analyzed the relevance between HMs and impacting factors, containing nonlinear relationships and linear relationships, but coefficient of Pearson correlation was not obviously, meaning that there was no obviously linear relationship between HMs and impacting factors, while it does not mean there was no non-linear relationship^51^. The influence of slope (X_5_) and aspect (X_6_) on HMs was not obvious, which may be due to the little variation range of aspect and slope in research area. The land use type (X_19_) only has significant impact on the As, Cr and Hg spatial distribution, but has no significant impact on the other 5 HMs spatial distribution.

Among the 11 distance factors X_7_, X_8_, X_9_, X_10_, X_11_, X_12_, X_13_, X_14_, X_15_, X_16_ and X_17_, except for X_14_ and X_16_, all have obvious influence on the spatial distribution of one or more HMs in soil of research region, it was represented in the complex impact of anthropogenic effect about the spatial distribution changes of soil HMs. Anthropogenic activities have shifted natural state of soil HMs distribution characteristics, creating fresh spatial characteristics^48^. Many studies have proved that there was an obvious enrichment of HMs in the soil around roads^52–54^or railways^55–57^ because the particles containing HMs formed by automobile exhaust emissions and tire wear enter the soil by atmospheric migration and deposition. River system was an significant water source for agricultural irrigation. HMs enrichment in water sources was caused through industrial discharge and transportation, and then farmland soil enrichment was caused through agricultural irrigation^58–60^. The HMs carried by the “three wastes” from various industries distributed in the study area were enriched in soil by atmospheric deposition, rain water infiltration and erosion. Related researches have displayed residential regions were the most frequent areas of anthropogenic activities, and daily life of residents will generate massive domestic waste containing HMs, which will lead to changes in soil around the residential regions, and the impact will also decrease with the increase of the distance from the residential areas^61,62^. At the same time, the dense traffic network and frequently anthropogenic activities in towns and cities makes massive pollutants enrich into soil by means of atmospheric deposition and other diffusion ways to produce pollution^63^.

## Conclusions

An integrated approach consists of multivariate statistical analyses, PCA model and GD model is an effective method to identify the ecological risk and sources of HMs in typical purple soil.The average Cd and Hg contents were lager than the Sichuan province soil background values. Mean Cr, Cd, Cu, Ni and Hg contents were lager than the purple soil background values. The ranking of I_geo_ evaluation results was Cd > Hg > Cr > Ni > Cu > Pb > Zn > As, the enrichment sequence of HMs in purple soil was Cd > Hg > Cr > Cu > Ni > Pb > Zn > As, the contamination sequence was Cd > Cu > Cr > Ni > Zn > Pb > As > Hg and the comprehensive pollution factor results show that there was no significant pollution of HMs in the soil of the research region. The sequence of potential ecological risks was Hg > Cd > As > Cu > Ni > Pb > Cr > Zn. Cd and Hg have the highest potential ecological risks and were at a higher risk level. PCA analysis show that Hg, As, Pb and Cd come from human activities, but Ni, Zn and Cr mainly come from natural sources, while Cu was affected by both natural sources and human activities. Geographical exploration analysis showed that, among the 25 influencing factors, X_18_, X_4_, TN, X_3_, X_2_ and X_1_ had the strongest explanatory power to explain the spatial differentiation of 8 HMs. This study provides useful information for determining the distribution characteristics, possible sources, and environmental risks of HM pollution in typical purple soil, which will help to develop targeted policies and measures to reduce HM pollution in purple soil environments.

## Data Availability

The authors declare that all data supporting the findings of this study are available within the article.
